# Partnering for enhanced digital surveillance of influenza‐like disease and the effect of antivirals and vaccines (PEDSIDEA)

**DOI:** 10.1111/irv.12645

**Published:** 2019-06-06

**Authors:** Barbara Rath, Helena C. Maltezou, Vassiliki Papaevangelou, Maria‐Alexandra Papagrigoriou‐Theodoridou, Maren Alchikh, Puja Myles, Brunhilde Schweiger, the PEDSIDEA Network, Hara Asimaki, Dimitra Dimopoulou, Christian Hoppe, Maria Karalexi, Kassiani Kekkou, Athanasios Kossivakis, Christine Kottaridi, Andreas Mentis, Ilia Vaki

**Affiliations:** ^1^ Vienna Vaccine Safety Initiative Berlin Germany; ^2^ Department of Epidemiology and Public Health The University of Nottingham School of Medicine Nottingham UK; ^3^ Department for Interventions in Healthcare Facilities Hellenic Centre for Disease Control and Prevention Athens Greece; ^4^ Third Department of Paediatrics University General Hospital ‘Attikon’, National Kapodistrian University of Athens Athens Greece; ^5^ First Department of Paediatrics Aghia Sophia Children's Hospital, National Kapodistrian University of Athens Athens Greece; ^6^ Department of Paediatrics Charité University Medical Centre Berlin Germany; ^7^ National Reference Centre for Influenza Robert Koch Institute Berlin Germany

**Keywords:** children, disease severity, ILI, influenza, mobile health, standardization

## Abstract

**Background:**

Standardised clinical outcome measures are urgently needed for the surveillance of influenza and influenza‐like illness (ILI) based on individual patient data (IPD).

**Objectives:**

We report a multicentre prospective cohort using a predefined disease severity score in routine care.

**Patients/Methods:**

The Vienna Vaccine Safety initiative (ViVI) Disease Severity Score (“ViVI Score”) was made available as an android‐based mobile application to three paediatric hospitals in Berlin and Athens between 2013 and 2016. Healthcare professionals assessed ILI patients at the point of care including severity, risk factors and use of antibiotics/antivirals/vaccines. RT‐PCR for influenza A/B viruses was performed at the Hellenic Pasteur Institute and the Robert Koch Institute. PCR testing was blinded to severity scoring and vice versa.

**Results:**

A total of 1615 children aged 0‐5 years (54.4% males) were assessed at the three sites. The mean age was 1.7 years (SD 1.5; range 0‐5.9). The success rate (completion of the scoring without disruption to the ER workflow) was 100%. ViVI Disease Severity Scores ranged from 0 to 35 (mean 13.72). Disease severity in the Berlin Cohort was slightly higher (mean 15.26) compared to the Athens Cohorts (mean 10.86 and 11.13). The administration of antibiotics was most prevalent in the Berlin Cohort, with 41.2% on antibiotics (predominantly cefuroxime) as opposed to only 0.5% on neuraminidase inhibitors. Overall, Risk‐adjusted ViVI Scores were significantly linked to the prescription of both, antibiotics and antivirals.

**Conclusions:**

The Risk‐adjusted ViVI Score enables a precision medicine approach to managing ILI in multicentre settings. Using mobile applications, severity data will be obtained in real time with important implications for the evaluation of antiviral/vaccine use.

## 1 INTRODUCTION

Acute respiratory viral infections and influenza‐like illness (ILI) are among the most common reasons for primary care visits and hospitalizations in children. Traditionally, hospitalization and admission to intensive care units have been considered criteria for “severe disease,” but clinical management decisions may differ from site to site. The European Respiratory Society emphasized that clinical outcomes, in particular mortality and hospitalization rates due to respiratory illness, vary significantly across Europe.^1^ For example, mortality appears to be higher in Eastern Europe, for reasons yet unknown.^1^ Improved understanding of regional differences will require validated, standardized disease severity measures.^2^ Standardized severity measures will allow cross‐cohort comparison and a precision medicine approach to managing individual influenza infections in different risk groups.^3^ Quality improvement programmes focused on optimizing treatment and prevention efforts depending on a patient's individual status will benefit from timely diagnostics and consistent use of standardized measures and operating procedures.^4^ Severity measures thus must be sufficiently granular to capture disease progression in patients with very mild to very severe disease, including within the intensive care unit.^5^

We present the first multicentre quality improvement programme implementing a standardized clinical severity measure for influenza‐like illness in routine care. QI efforts are designed to induce system‐level change. The participating departments agreed to introduce an institution‐wide standard operating procedure, which is implemented in specific case scenarios (in this case, ILI) with regular analysis and evaluation. The PEDSIDEA operating procedure was introduced into routine care as a “standing order” for predefined standardized disease severity assessments and diagnostic testing in all patients with ILI, regardless of the reason for presentation. The Vienna Vaccine Safety initiative (ViVI) Disease Severity Score was made available via mobile application in three different paediatric hospitals and two reference laboratories in Germany and Greece (Partnering for Enhanced Digital Surveillance of Influenza‐like Disease and the Effect of Antivirals and Vaccines: PEDSIDEA).

## 2 METHODS

### 2.1 Severity assessments

As reported previously, the ViVI Disease Severity Score is a standardized clinical outcome measure that can be used independent of clinical treatments or interventions. The ViVI Score mobile application provides a uniform approach to defining *ad hoc* disease severity at any given time point, based on extensive literature review as well as WHO Criteria for uncomplicated and complicated influenza.^6^ The ViVI Score consists of nine unweighted symptoms/items reflecting uncomplicated disease (DSU1‐9) plus 13 weighted items reflecting complicated disease (DSC 1‐13) resulting in overall scores ranging from 0‐48.^2^, ^3^, ^5^ Data formats and terminologies are fully compliant with Clinical Data Interchange Standards Consortium (CDISC) and regulatory requirements.^3^

For validation in a multicentre quality improvement (QI) programme, the ViVI Score was made available as a mobile application for android systems, linked to a central database. The ViVI Score App (https://score.vi‐vi.org) was provided by the Vienna Vaccine Safety Initiative to three academic children's hospitals: Charité University in Berlin Germany, Aghia Sophia Children's Hospital and University General Hospital “Attikon” (ie, 1st and 3rd Departments of Paediatrics) at Kapodistrias University Athens, Greece.^7^ The programme was approved by the respective institutional review boards (Charité: EA24/008/10; Attikon: 483/05‐11‐2014, Aghia Sophia: 27509/2‐12‐2014). Informed consent procedures were waived for the purpose of enhanced diagnostics and quality of care. At each site, monitoring throughout two consecutive influenza seasons (January‐May of the same year from 2014 to 2016) was required.

The severity assessments were performed by independent QI staff in patients with influenza‐like illness (ILI), at the time of initial presentation to the emergency room (ER)/hospital, that is prior to any treatment decisions.^2^ Assessments included the ViVI Disease Severity Score, the ViVI Risk Factor Score (consisting of 16 unweighted items^3^) and three simple yes/no questions regarding planned treatment with antibiotics and/or antivirals and the patient's current flu vaccination status. The calculation of the ViVI Disease Severity Score, the number of risk factors and the Risk‐adjusted ViVI Score are listed in the Supporting Information.

### 2.2 Virology

RT‐PCR for influenza A/B viruses was performed at the Hellenic Pasteur Institute, Attikon Hospital and the Robert Koch Institute: At the National Influenza Centre in Berlin, nasopharyngeal swabs were received and eluted in 3.0 mL cell culture medium. After RNA extraction and cDNA synthesis, real‐time PCR was performed using Light Cycler 480 real‐time PCR system. Primer and probes for amplification as well as typing and subtyping were used as described recently.^8^ At the Attikon Hospital laboratory, RNA was extracted with QIAamp Viral RNAmini (Qiagen) using the QIAcube technology for automated extraction. All specimens were analysed to assess the quality of the specimen and extraction procedure, as well as for the presence of influenza virus by real‐time RT‐PCR with primers and probes as described in WHO molecular diagnostic protocols.^9^ Aghia Sophia samples were analysed at the Hellenic Pasteur Institute^10^ using NucliSENS® easyMAG® platforms (bioMérieux Hellas) and an in‐house multiplex real‐time RT‐PCR. The PCR protocol is validated according to ISO 15189 requirements and deposited with the European Influenza Surveillance Network.^11^ Virological laboratories were blinded to ViVI Scores, and influenza PCR results were made available after patient discharge, that is after severity scoring was completed and uploaded.

### 2.3 Data analysis

Descriptive statistics (percentages, summary measures and histograms) were used to map the distribution of the ViVI Disease Severity Score (ViVI Score) and risk factors (ViVI Risk Factor Score, RF‐Score) across the three PEDSIDEA sites. The correlation between RF‐Scores, the VIVI Scores and treatment decisions was assessed using mean differences and *t* tests to assess significance. Pearson's correlation coefficient was used to assess the correlation between the RF‐scores and age. Finally, the ViVI Score/RF‐Score Index was developed to take into account both disease severity and pre‐existing risk factors so as to better predict patient outcomes (see Supporting Information). All analyses were conducted using stata version 14.

## 3 RESULTS

### 3.1 Population and demographics

**Figure 1:**
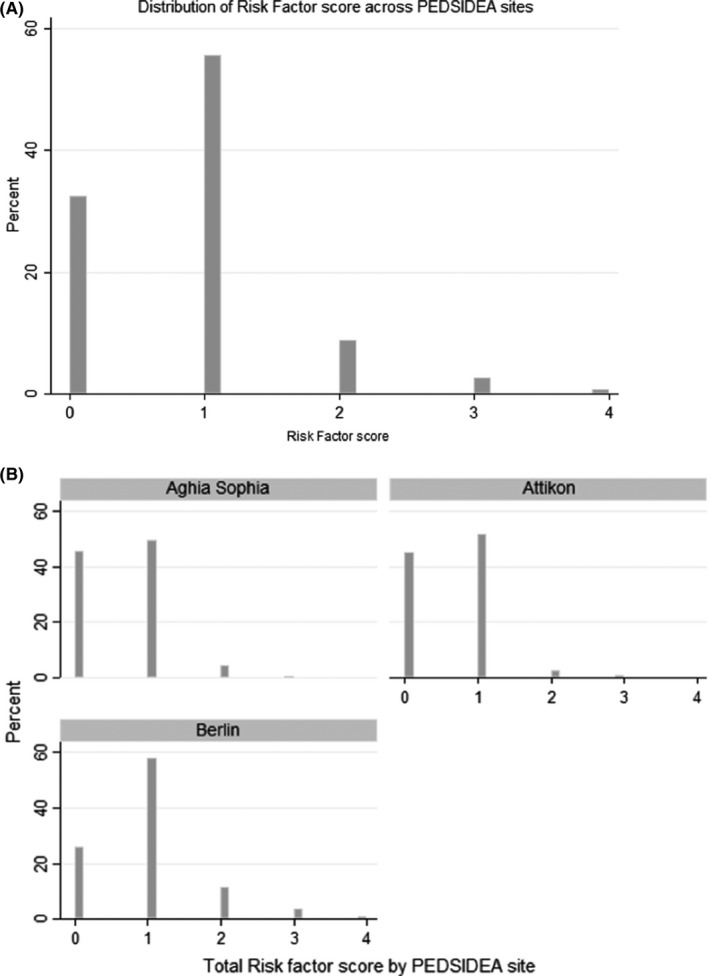
Distribution of ViVI Risk Factor Scores (A) over the PEDSIDEA cohort (n = 1615) (B) by PEDSIDEA site. PEDSIDEA, Partnering for Enhanced Digital Surveillance of Influenza‐like Disease and the Effect of Antivirals and Vaccines. ViVI, Vienna Vaccine Safety initiative

Between 1 January 2013 and 31 May 2015, a total of 1615 children aged 0‐5 years from three hospital sites (two in Athens and one in Berlin) were included in the QI programme and analysis. The success rate (completion of the scoring without disruption to the ED workflow) was 100%. The mean age was 1.7 years (SD 1.5; range 0‐5.9), and the median age (IQR) was 1.3 (0.5‐2.7) years for the overall PEDSIDEA cohort, and there were 54.4% males. The mean RF‐Score was 0.86 (SD 0.74, range 0‐4) given a maximum possible RF‐Score of 16, while the median RF‐Score (IQR) was 1 (0‐1). The demographic characteristics and distribution of risk factors for the overall cohort and by study site are summarised in Table 1, while the distribution of RF‐Scores is plotted in Figure 1A,B.

**Table 1:** Patient demographic characteristics, risk factors and influenza status (n = 1615)

Patient characteristic or risk factor (RF)	Berlin (n = 1030)	Aghia Sophia (n = 285)	Attikon (n = 300)	PEDSIDEA (n = 1615)
Age in years (mean; range)	1.6 (0‐5.9)	1.7 (0‐5.7)	2.1 (0.04‐5.8)	1.7 (0‐5.9)
Gender (males)	562 (54.6%)	151 (53.0%)	165 (55.0%)	878 (54.4%)
RF1: Infant under 2 y	717 (69.6%)	147 (51.6%)	151 (50.3%)	147 (51.6%)
RF2: Pulmonary condition	68 (6.6%)	7 (2.5%)	12 (4.0%)	87 (5.4%)
RF3: Cardiac condition	106 (10.3%)	0 (0.0%)	0 (0.0%)	106 (6.6%)
RF4: Diabetes	3 (0.3%)	0 (0.0%)	0 (0.0%)	3 (0.2%)
RF5: Obesity	1 (0.1%)	0 (0.0%)	1 (0.3%)	2 (0.1%)
RF6: Other metabolic disease	23 (2.2%)	1 (0.4%)	1 (0.3%)	25 (1.6%)
RF7: Chronic renal disease	24 (2.3%)	2 (0.7%)	0 (0.0%)	26 (1.6%)
RF8: Chronic hepatic disease	10 (1.0%)	1 (0.4%)	0 (0.0%)	11 (0.7%)
RF9: Neurologic condition	57 (5.5%)	9 (3.2%)	8 (2.7%)	74 (4.6%)
RF10: Haemoglobinopathies	11 (1.1%)	1 (0.4%)	1 (0.3%)	13 (0.8%)
RF11: Congenital immunosuppression	2 (0.2%)	1 (0.4%)	0 (0.0%)	3 (0.2%)
RF12: Acquired immunosuppression	27 (2.6%)	0 (0.0%)	3 (1.0%)	30 (1.9%)
RF13: Aspirin therapy	18 (1.8%)	1 (0.4%)	0 (0.0%)	19 (1.2%)
RF14: Pregnancy	0 (0.0%)	0 (0.0%)	0 (0.0%)	0 (0.0%)
RF15: Elderly	0 (0.0%)	0 (0.0%)	0 (0.0%)	0 (0.0%)
RF16: Prematurity	65 (6.3%)	12 (4.2%)	20 (6.7%)	97 (6.0%)
Total ViVI Risk Factor Score (mean; range)	**1.01 (0‐4)**	**0.60 (0‐3)**	**0.59 (0 −3)**	**0.86 (0‐4)**
Confirmed influenza infection	**114 (11.1%)**	**138 (48.4%)**	**99 (33.0%)**	**351 (21.7%)**
Influenza A	99 (9.6%)	119 (41.8%)	90 (30.0%)	308 (19.1%)
Influenza B	15 (1.5%)	19 (6.7%)	9 (3.0%)	43 (2.7%)

Abbreviation(s): ViVI, Vienna Vaccine Safety initiative.

### 3.2 ViVI Disease Severity Score

**Figure 2:**
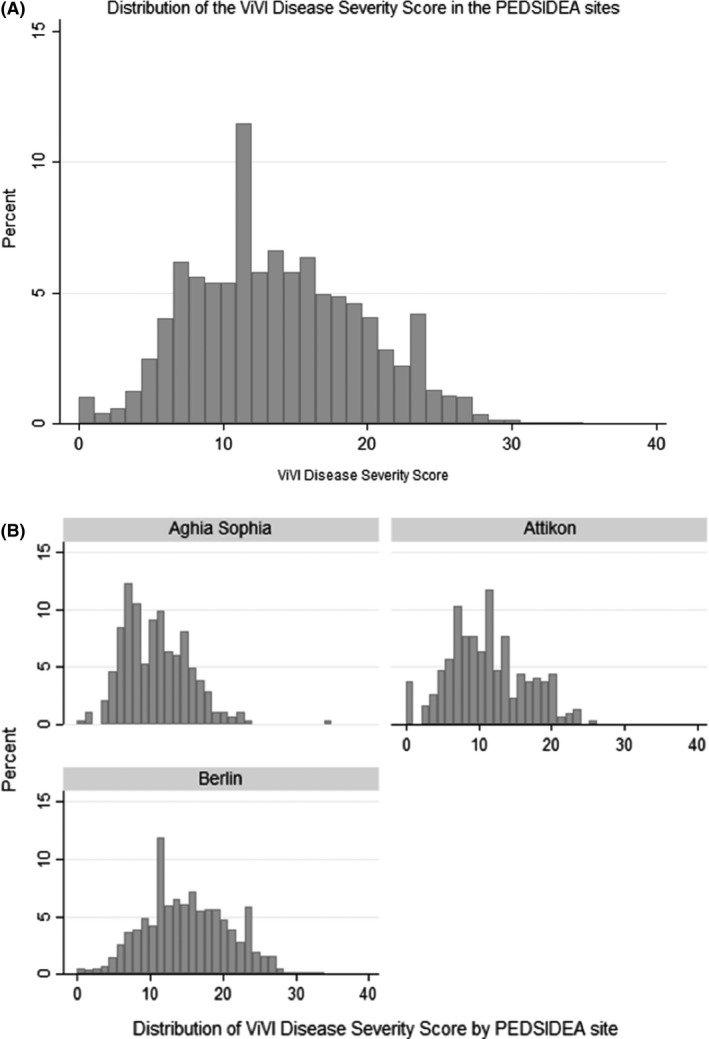
Distribution of ViVI Disease Severity Scores (A) over the PEDSIDEA cohort (n = 1615) (B) by PEDSIDEA site. PEDSIDEA, Partnering for Enhanced Digital Surveillance of Influenza‐like Disease and the Effect of Antivirals and Vaccines. ViVI, Vienna Vaccine Safety initiative

The mean ViVI Score was 13.72 (SD 5.81; range 0‐35) given a possible maximum score of 48, while the median score (IQR) was 14 (9‐18). Figure 2A,B plots the distribution of the VIVI Score for the whole cohort and by study site. Table 2 summarises the individual disease severity criteria in the PEDSIDEA cohort.

**Table 2:** ViVI Disease Severity Score criteria and treatment decisions (n = 1615)

ViVI Score item/Prescribing practice	Berlin (n = 1030)	Aghia Sophia (n = 285)	Attikon (n = 300)	Overall PEDSIDEA (n = 1615)
DSU 1: Fever	868 (84.3%)	253 (88.8%)	239 (79.7%)	1360 (84.2%)
DSU 2: Cough	214 (71.3%)	244 (85.6%)	255 (85.0%)	1333 (82.5%)
DSU 3: Pharyngitis	459 (44.6%)	178 (62.5%)	214 (71.3%)	851 (52.7%)
DSU 4: Coryza/Rhinitis	799 (77.6%)	207 (72.6%)	268 (89.3%)	207 (72.6%)
DSU 5: Headache	34 (3.3%)	13 (4.6%)	20 (6.7%)	67 (4.2%)
DSU 6: Myalgia	13 (1.3%)	12 (4.2%)	12 (4.0%)	37 (2.3%)
DSU 7: Malaise	263 (25.5%)	57 (20.0%)	199 (66.3%)	519 (32.1%)
DSU 8: Diarrhoea	52 (17.3%)	42 (14.7%)	52 (17.3%)	285 (17.7%)
DSU 9: Vomiting	330 (32.0%)	50 (17.5%)	55 (18.3%)	435 (26.9%)
DSC 1: High and prolonged fever	97 (9.4%)	33 (11.6%)	22 (7.3%)	152 (9.4%)
DSC 2: Dyspnoea	499 (48.5%)	84 (29.5%)	75 (25.0%)	658 (40.7%)
DSC 3: Hypoxia	304 (29.5%)	16 (5.6%)	38 (12.7%)	358 (22.2%)
DSC 4: Haemoptysis	17 (1.7%)	3 (1.1%)	0 (0.0%)	20 (1.2%)
DSC 5: Altered or loss of consciousness	30 (2.9%)	12 (4.2%)	31 (10.3%)	73 (4.5%)
DSC 6: Seizure	108 (10.5%)	1 (0.4%)	0 (0.0%)	109 (6.8%)
DSC 7: Dehydration	110 (10.7%)	6 (2.1%)	11 (3.7%)	127 (7.9%)
DSC 8: Exacerbation of chronic disease	4 (0.4%)	0 (0.0%)	9 (3.0%)	13 (0.8%)
DSC 9: Septic shock or multiorgan failure	3 (0.3%)	5 (1.8%)	0 (0.0%)	8 (0.5%)
DSC 10: Need for hospitalisation	784 (76.1%)	156 (54.7%)	150 (50.0%)	1090 (67.5%)
DSC 11: Lower respiratory tract infection/super‐infection	881 (85.5%)	242 (84.9%)	235 (78.3%)	1358 (84.1%)
DSC 12: Upper respiratory tract infection/ super‐infection	467 (45.3%)	117 (41.1%)	96 (32.0%)	680 (42.1%)
DSC 13: Need for ICU admission	321 (31.2%)	5 (1.8%)	8 (2.7%)	334 (20.7%)
Total VIVI SCORE (mean; range)	**15.26 (0‐33)**	**10.86 (1‐35)**	**11.13 (0 −26)**	**13.72 (0‐35** **)**
Antivirals planned	2 (1.1%)	28 (9.8%)	53 (17.7%)	83 (10.9%)
Antivirals prescribed	1 (0.5%)	6 (2.1%)	45 (15.0%)	52 (6.8%)
Antibiotics planned	58 (31.7%)	64 (22.5%)	72 (24.0%)	214 (28.2%)
Antibiotics prescribed	84 (41.2%)	21 (7.4%)	66 (22.0%)	171 (21.7%)

Abbreviation(s): ViVI, Vienna Vaccine Safety initiative.

### 3.3 Prescribing practices across PEDSIDEA sites

Oseltamivir was the preferred antiviral across all three sites (Table 2). The most commonly used antibiotic class across the three sites was cephalosporins (cefotaxime, cefuroxime, ceftriaxone and cefprozil) followed by penicillins (amoxicillin, ampicillin, penicillin and amoxicillin + clavulanate). In a few cases, antibiotic combinations were prescribed which included vancomycin, erythromycin, azithromycin, ciprofloxacin, metronidazole or gentamicin in addition to a cephalosporin or penicillin. There appeared to be a slight preference for using cephalosporins in the Berlin site as compared to the two Athens sites.

### 3.4 Association between ViVI Risk Factor Score and treatment decisions in the ER, as well as reported antibiotic/antiviral pre‐exposures

No significant difference in total risk factor score (RF‐Score) was observed for cases where antiviral treatment was planned at the time of scoring and presentation to the ER (0.14 [95% CI: −0.02 to 0.31]; *P* = 0.0866). The RF‐Score was slightly lower in those cases where antibiotic treatment was planned at the time of presentation (0.13 [95% CI: 0.01‐0.25]; *P* = 0.0271).

No significant difference was observed in the mean total RF‐Score among those who had reported any previous prescription of antibiotics during the same disease episode (0.06 [95% CI: −0.05 to 0.18]; *P* = 0.2857) or antivirals (0.14 [95% CI: −0.06 to 0.35]; *P* = 0.1570).

### 3.5 Correlation of ViVI Disease Severity Score with treatment decisions

Vienna Vaccine Safety initiative Scores at the time of presentation were not significantly correlated with physicians' plans for antiviral treatment (mean ViVI Score in patients where antivirals were planned was 14.02 [95% CI: 12.94‐15.11] vs 13.70 [95% CI: 13.41‐13.99]; *P* = 0.6191). The mean ViVI Score was, however, significantly associated with planned antibiotic treatment (mean ViVI Score in patients where antibiotics were planned was lower at 11.47 [95% CI: 10.69‐12.24] as compared to that in patients where antibiotic treatment was not planned: 13.99 [95% CI: 13.69‐14.29]; *P* < 0.001).

Patients who had reported a previous antiviral prescription had non‐significant, slightly higher mean ViVI Disease Severity Scores (15.19; 95% CI: 13.89‐16.49) as compared to those who did not report a previous antiviral prescription (13.67; 95% CI: 13.38‐13.95); *P* = 0.0604.

A borderline significant difference was observed in those who had reported past antibiotic prescriptions (14.54; 95% CI: 13.64‐15.43) as compared to those who had not (13.62; 95% CI: 13.32‐13.92); *P* = 0.0501.

### 3.6 Correlation of ViVI Disease Severity Score with influenza infection

Patients with confirmed influenza had a significantly lower mean ViVI Disease Severity Scores than those without influenza ([11.15; 95% CI: 10.57‐11.73] and [14.45; 95% CI: 14.13‐14.76] respectively; *P* < 0.001).

Among influenza patients, there was no significant difference in mean ViVI Scores in patients who had received a seasonal influenza vaccination (−2.28; 95% CI: −7.15 to 2.59); *P* = 0.3575 and those who had not received a seasonal influenza vaccination (−0.10; 95% CI: −2.50 to 2.31); *P* = 0.9354. It should be noted, however, that only 5/351 (1.4%) influenza positive cases had received seasonal influenza vaccination and 22/1255 (1.8%) patients without influenza had received seasonal influenza vaccination.

### 3.7 Correlation of ViVI Disease Severity Score with the ViVI Risk Factor Score

There was a significant but weakly positive correlation between the RF‐Score and the ViVI Score (Pearson's correlation coefficient 0.2404; *P* < 0.001).

### 3.8 Risk‐adjusted ViVI Score: a new score based on disease severity and patient risk factors to predict patient outcomes and need for treatment

**Figure 3:**
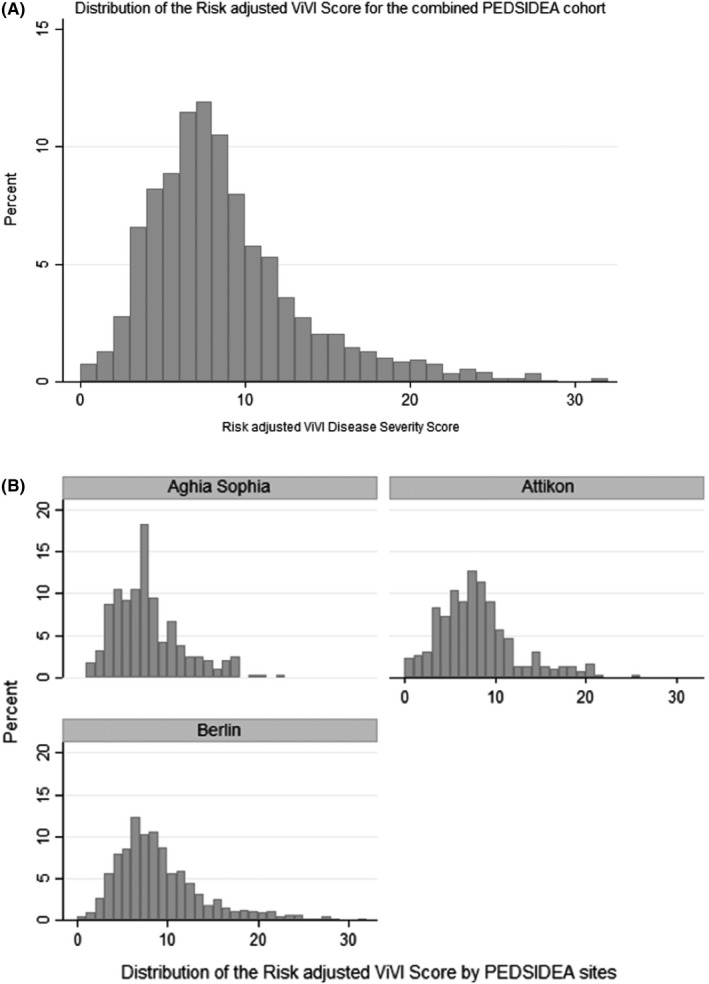
Distribution of Risk‐adjusted ViVI Score (A) over the PEDSIDEA cohort (n = 1615) (B) by PEDSIDEA site. PEDSIDEA, Partnering for Enhanced Digital Surveillance of Influenza‐like Disease and the Effect of Antivirals and Vaccines. ViVI, Vienna Vaccine Safety initiative

The mean Risk‐adjusted ViVI Score was 8.29 (SD 4.56; range 0‐32), while the median (IQR) was 7.5 (5‐10). Figure 3A,B shows the distribution of the Risk‐adjusted ViVI Score for the overall PEDSIDEA cohort and by study site.

Patients who received antiviral treatment had a significantly higher Risk‐adjusted ViVI Score than those who did not receive antivirals (mean Risk‐adjusted ViVI Score of 10.25; 95% CI: 8.64‐11.85 as compared to 8.22; 95% CI: 8.00‐8.44 respectively; *P* = 0.0015). Similarly, a significant difference was observed in mean Risk‐adjusted ViVI Scores for patients who received antibiotics as compared to those who did not receive antibiotics (mean Risk‐adjusted ViVI Score of 9.21 [95% CI: 8.39‐10.02] vs 8.18 [95% CI: 7.95‐8.41]; *P* = 0.0051).

### 3.9 Distribution of Risk‐adjusted ViVI Score by age and by viral aetiology

Pearson's correlation coefficient *r* = 0.3323; *P* < 0.001 shows a significant but weak positive correlation between age and Risk‐adjusted ViVI Score (Supporting Information).

The mean Risk‐adjusted ViVI Score in patients with confirmed influenza was 8.00 (95% CI: 7.50 to 8.50) as compared to 8.37 (95% CI: 8.12‐8.61) in patients without influenza (*P* = 0.1838), that is no significant difference in mean Risk‐adjusted ViVI Scores by influenza status.

## 4 DISCUSSION

This is the first report of the use of the ViVI Score in a paediatric multicentre setting in Europe. The PEDSIDEA Network proof‐of‐concept project demonstrated that shared scoring systems via mobile applications enable the real‐time surveillance of influenza disease severity. No major training was required for medical staff to use the mobile application for instantaneous data acquisition allowing comparison of disease presentation, influenza epidemiology and patient management across sites. The Risk‐adjusted ViVI Score was highly predictive of physician prescribing practice with regard to antibiotics and antivirals indicating possible use in antibiotic stewardship and quality improvement programmes. Ideally, the severity scoring should be combined with virus diagnostics, as was done here in collaboration with the Robert Koch Institute and the Hellenic Pasteur Institute.

The present study shows an important advancement in relation to what was published previously. Patients with comparable levels of severity do not always receive the same treatment, as seen in the three hospitals. The ViVI Score however, can be used to understand physician behaviour and differences in the handling of respiratory viral infections across Europe and beyond.

The ViVI Disease Severity Score is designed to ensure that the same data are collected at the point of care, that is at the time when the patient is in front of the assessor, regardless of the setting. As a symptom‐based score, it does not require access to laboratory or imaging facilities. The use of mobile technology ensures that data entry is accompanied by the assessor's (ie, healthcare provider's) user ID, audit trail, geomapping, and time stamps. Once data are entered and transmitted, the score cannot be modified. Use of this simple tool improves data integrity, minimizes observer bias and eliminates missing data. The ViVI Scorecore can be calculated for each patient individually, providing real‐time assessments at the time of initial presentation, or during follow‐up visits.^3^ This level of standardization allows studying differences in patient populations and management, that is how patients with comparable levels of disease severity are managed in different settings.

Uniform outcome measures will facilitate the comparison of medical interventions in multicentre clinical trials and post‐marketing surveillance. Regulatory authorities in Europe and North America have called for standardized clinical outcome measures to facilitate the systematic evaluation of antiviral drugs.^1^, ^12^, ^13^, ^14^ WHO priorities, at the same time, indicate that next‐generation influenza vaccines should mostly prevent severe disease outcomes. Again, standardised scores will be needed for the monitoring of influenza and RSV vaccine effectiveness.^15^ The ViVI Disease Severity Score is based on WHO criteria for uncomplicated and complicated disease^6^ and extensive review of the literature covering clinical trials and observational studies of influenza and other respiratory viral infections.^3^

The ViVI Score may be used to promote antibiotic stewardship.^2^ Severity scoring in conjunction with reviews of immunization records and targeted bacterial cultures significantly can reduce the inappropriate use of antibiotics and thus cost.^2^ Standardized severity assessments in high‐risk patients, combined with rapid diagnostics, could help to facilitate early treatment at the time of maximum effectiveness.^14^, ^16^, ^17^ Additional innovation was introduced with the Risk‐adjusted ViVI Score, which was closely linked to antibiotic use. The fact that the treating physicians were unaware of the Score at the time of treatment decisions indicates that a higher Risk‐adjusted ViVI Score reflects a perceived “need to do something.” If a patient is perceived as sick “out of proportion” for their assumed risk factor profile, doctors may feel the urge to use antibiotics, even though ineffective in respiratory viral infections.

The project had several strengths and limitations: The evaluation was limited to three urban tertiary care centres in Europe. More research is needed in adults, in remote settings, and in populations with limited resources. The ViVI Score mobile app does not rely on handwritten clinical notes or uneven electronic recording based on a variety of coding approaches. Existence of standardized outcome assessments greatly facilitates international collaboration and meta‐analyses. Large‐scale international studies of disease burden are warranted before new programmes are rolled out.^18^, ^19^, ^20^

Future studies will include longitudinal components allowing the assessment of treatment effects over time. Similarly, vaccine effectiveness will be studied in settings where influenza vaccination is universally recommended in children, unlike in Germany and Greece, where no such recommendation has been issued and where paediatric vaccination rates are low. (see www.keelpno.gr for Greece and https://bit.ly/2C0FFUd for Germany). For further optimisation, PEDSIDEA samples should be handled by one central laboratory or using one diagnostic method. The current study focused on influenza. The role of other viral and bacterial pathogens was not assessed. It was suggested that co‐infections have little impact, or elicit less severe disease compared to monoinfections.^21^ Previous analyses using the ViVI Score were inconclusive 2, 3 and require further investigation in multicentre settings.

This paper aims to present a simple, standardized way of measuring clinical outcomes in children at the time of initial presentation to allow for meaningful comparisons across clinical settings. It invites other clinicians to use these standardized measures as well, to improve the monitoring of quality of care, to understand overall disease burden and the prevalence of risk factors, and finally, to explore the relationship between severity and prescribing practices, cost, and other outcome measures of interest.

The successful PEDSIDEA pilot programme demonstrates that surveillance systems for influenza can be set‐up quickly enabling individualized patient data analysis in epi/pandemics.^3^, ^22^ Stakeholders will receive real‐time information on influenza incidence and severity, allowing the allocation of resources where they are most in need. This is important as newly emerging influenza viruses may transmit poorly while eliciting considerable disease severity.^23^ Current surveillance systems are focused on numbers and mortality^24^ but may be missing severe non‐lethal disease. Our current knowledge is limited with respect to possible mechanisms underlying severe outcomes with influenza infection.^25^, ^26^ Standardized scoring systems will be key to the identification of virus and host factors related to severe outcomes.^27^, ^28^ Validated biomarkers predicting severity will assist future physicians in tailoring therapies to their patients’ individual needs.

The ViVI Score App (https://score.vi-vi.org) provides a useful instrument to harmonize severity assessments in multicentre clinical trials and observational studies. Future studies will explore use of the ViVI Score App in adult patients and for patient/parent‐reported outcomes. Interesting differences have been observed between sites: patients with the same level of severity did not necessarily receive the same treatment. Standardization will provide a useful path forward with important implications for best practice and policy.

## 5 CONCLUSIONS

The Risk‐adjusted ViVI Score allows the consistent measurement of disease severity in urgent care and multicentre settings. The significance of the Risk‐adjusted ViVI Score indicates that physicians may be more likely to resort to antibiotics or antivirals if they perceive a patient as “too ill” in relation to the number of risk factors. Standardized risk factor data and severity data have important implications for influenza surveillance and the critical evaluation of antibiotic and antiviral use, as well as vaccine effectiveness.^29^ Surveillance programmes are strengthened enabling public health authorities to detect highly pathogenic viruses early on, even if they are prevalent at low rates.^30^ Future studies will include clinical trials, adult ILI surveillance studies, and the alignment of patient‐ and physician‐reported outcome measures.

## APPENDIX 1

### PEDSIDEA NETwoRK GROUP AUTHORSHIPS

Hara Asimaki, MD, Aghia Sophia Children's Hospital, Kapodistrias University, Athens, Greece; Dimitra Dimopoulou MD, University General Hospital ATTIKON, Kapodistrias University, Athens, Greece; Christian Hoppe BS, Vienna Vaccine Safety Initiative, Berlin, Germany; Maria Karalexi MD, University General Hospital ATTIKON, Kapodistrias University, Athens, Greece; Kassiani Kekkou MD, University General Hospital ATTIKON, Kapodistrias University, Athens, Greece; Athanasios Kossivakis MS, Hellenic Pasteur Institute, Athens, Greece; Christine Kottaridi PhD, University General Hospital ATTIKON, Kapodistrias University, Athens, Greece; Andreas Mentis, MD, Hellenic Pasteur Institute, Athens, Greece; Ilia Vaki MD, Aghia Sophia Children's Hospital, Kapodistrias University, Athens, Greece.
