# Supplementary material for: Partnering for enhanced digital surveillance of influenza‐like disease and the effect of antivirals and vaccines (PEDSIDEA)
Source: Influenza Other Respir Viruses. 2019 Jun 6;13(4):309–18. doi: 10.1111/irv.12645 (PMC6586183; doi:10.1111/irv.12645)
Supplement: Supplementary file 1 [file IRV-13-309-s001.docx]

**The ViVI Disease Severity Score** ****

**The Number of Risk Factors**

*Source: B Rath, T Conrad, P Myles, M Alchikh, X Ma, C Hoppe, F Tief, X Chen, P Obermeier, B Kisler, B Schweiger: Influenza and other respiratory viruses: standardizing disease severity for surveillance and clinical trials. Expert Review of Anti-Infective Therapy 2017, 15(6):545-568*

**The Risk-adjusted ViVI-Score**

**ViVI Disease Severity Score / (ViVI Risk Factor Score +1)**

**Correlation between age and Risk-adjusted ViVI-Score**

**
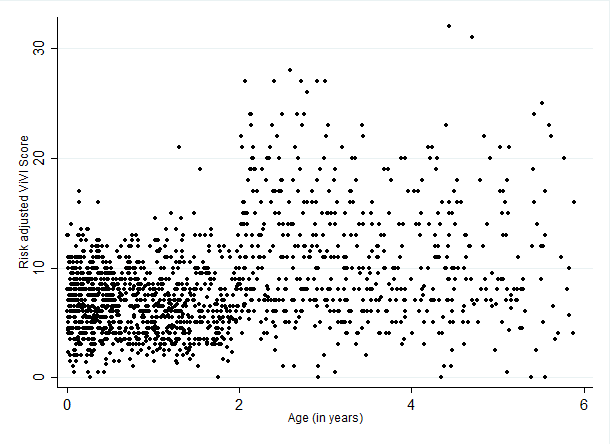
**
